# A national survey of caregiver’s own experiences and perceptions of U.S. health care system when addressing their health and caring for an older adult

**DOI:** 10.1186/s12913-021-06086-z

**Published:** 2021-01-29

**Authors:** Jill C. Slaboda, Sandahl H. Nelson, Zia Agha, Gregory J. Norman

**Affiliations:** 1 Gary and Mary West Health Institute, 10350 N. Torrey Pines Road, La Jolla, CA 92037 USA; 2 Department of Family Medicine and Public Health, University of California San Diego, 9500 Gilman Drive, La Jolla, CA 92093 USA

**Keywords:** Family caregivers, Access to health care, Caregiver support, Older adult, And community care

## Abstract

**Background:**

Caregiving is a demanding role that can negatively impact a person’s health and well-being. As such, adequate access to health care is important for maintaining the family caregiver’s own personal health. The aims of this study were to identify if family caregivers of older adults had more difficulty accessing health care services than non-caregivers and to identify if family caregivers felt access to additional services would be beneficial for maintaining their own personal health care.

**Methods:**

National survey of 3026 US adults aged 30 to 89 years old. Participants were grouped based on self-reported caregiving experience. Survey asked about access to care, importance of health care services and whether caregivers had support needed. Descriptive statistics were used to compare caregiver and non-caregiver’s responses. Multivariate logistic regression model assessed correlates of caregivers not having the support they needed.

**Results:**

Caregivers were older, female, lower educational attainment, lower income, had more multiple chronic health conditions and health condition or disability that impacts their daily life. Caregivers reported difficulty accessing mental health services, dental services, medications, and supportive services at home. Caregivers felt it was important to have care coordinator, long-term relationship with primary care provider and access to house calls, telemedicine, and medications delivered to the home. Age, ethnicity, chronic conditions and confidence in finances were factors influencing whether caregiver had support needed to provide assistance to older care recipient.

**Conclusion:**

Caregivers provide needed support and care to older adults while also needing support for themselves. Health care services delivered in the home were highly desirable to caregivers and could help them maintain their health and well-being.

## Background

Approximately 17.7 million people in the United States are serving as unpaid or family caregivers for an older adult with majority of these caregivers providing care to an older adult with significant health and/or functioning needs [1]. The role of caregiving can range from assistance with self-care and activities of daily living to assistance with complex medical tasks such as medication management, health care coordination and wound care [2–5]. Family caregivers are often supporting and attending to the health care needs of the care recipient while also needing support themselves [6]. Family caregivers are more likely to experience physical, emotional, and economic harm due to the burden of providing care [7–10]. The responsibilities of providing care to an older adult can extend over long periods of time and these responsibilities can expand as progression of chronic diseases and dementia slowly reduce functioning in the care recipient [11].

The physical and emotional aspects associated with caregiver burden will not only impact the caregiver’s health but can also have detrimental effects on the care recipient. Family caregivers who felt their role was emotionally and physically difficult were more likely to not meet the needs of their care recipient, suggesting high stress and burden can result in compromised care [12]. Similarly, family caregiver sadness and severe fatigue have been related to increased emergency department use and health care expenditures in care recipients who are functionally disabled [13]. In contrast, family caregivers whose burden and well-being were addressed through innovative medical and/or social support models that provide telephonic support, counseling, education and/or in-home health care have shown reduced health care expenditure and delay nursing home placement of care recipients [14–17].

Despite this evidence, systematic approaches and operational processes to support family caregivers for their own health and the health of their care recipient within the American health care system are still lacking [1, 18, 19]. Time constraints of primary care providers often prohibit the adequate assessment of the needs of patients and their caregivers, especially when patients have chronic illness [20]. Gaps in care coordination, access to care and lack of shared decision-making have been reported as missing aspects of health care encounters for patients with chronic illness and their caregivers [21, 22]. These gaps in healthcare delivery suggest caregivers’ access to health care and need for services are very different than their non-caregiving peers.

Access to health care has been defined in a conceptual framework as the opportunity to identify a health care need, seek health care services, reach, obtain or use health care services and have this need for services fulfilled [23]. This framework includes five dimensions of accessibility that are Approachability, Acceptability, Availability and accommodation, Affordability and Appropriateness and five corresponding abilities of the population that include ability to perceive, ability to seek, ability to reach, ability to pay and ability to engage [23]. This conceptual framework describes the complex interactions that influence access to health care from the health system, organization, institutional and provider perspective and the patient, population and community level perspective.

In this study, we used this conceptual framework with the aims of identifying if family caregivers of older adults had more difficulty accessing health care services than non-caregivers and to identify if family caregivers felt access to additional services would be beneficial for maintaining their own health care. Specifically, we investigated the caregiver’s perceived ability to reach, engage and seek health care services. Family caregivers were asked their perceived difficulty in obtaining health care services for their own health care needs through a national survey conducted in the United States. We assessed if existing models of care that have shown to increase access, such as coordinated care and health care services in the home [24–27], were important to family caregivers for addressing their own health care needs. As home-based care models and telehealth become more available [28], we assessed response from both caregivers and non-caregivers to understand whether access to health care and demand for health care services in the home were different. Finally, we asked caregiver whether they had enough support when caring for their care recipient. We hypothesize that caregivers will have greater difficulty accessing services for their own personal health and will be more likely to want services provided in the home compared to non-caregivers.

## Methods

### Participants

A cross-sectional research design was used in this study. Survey respondents were a general population sample of 3026 US adults aged 30 to 89 years from the National Opinion Research Center (NORC) AmeriSpeak® panel, which is a probability-based representative panel of civilian noninstitutionalized adults living in the United States of America. The sample for this study was selected using stratified sampling, with strata based on age, race/ethnicity, education, and gender. The final sample was made nationally representative by weighting. This weighting included base weights of the inverse probability of selection from the NORC frame, and then further adjusted to account for unknown eligibility and nonresponse among eligible participants. The survey was approved by the NORC Institutional Review Board (IRB). Participants were recruited from the AmeriSpeak® panel and gave permission to be part of the panel and contacted for the survey.

Surveys were conducted from September to October 2016, offered in English and Spanish, on both phone and web platforms, and took an average of 26 min to complete. The survey contained 51 questions and asked questions on aging perceptions and access to health care. Results of aging perceptions have been published elsewhere [29]. Some demographic information had been previously collected from participants as part of being in the AmeriSpeak® panel.

### Survey measures

Participants were grouped by self-reported history of being a caregiver. Specifically, participants were asked “Some people need ongoing living assistance as they get older. This assistance can help with things like keeping house, cooking, bathing, getting dressed, getting around, paying bills, remembering to take medicine, or just having someone check in to see that everything is okay. This help can happen at your own home, in a family member’s home, in a nursing home, or in a senior community. And, it can be provided by a family member, a friend, a volunteer, or a health care professional. Are you currently providing or have you ever provided ongoing assistance directly to an older family member or close friend?” Participants who answered ‘yes’ were labeled as caregivers and participants who answered ‘no’ were labeled as non-caregivers.

Demographic information about participant’s age, gender, ethnicity, educational level, employment status, whether they lived in metropolitan area, home ownership, if children were in the home, Medicaid status and marital status was collected. Participants were asked if they had any health conditions or physical disabilities that impact their daily life or limit their activities. Finally, participants were asked if they had any chronic conditions such as hypertension, coronary heart disease, stroke, chronic lung disease, cancer, diabetes, arthritis, hepatitis, weak or failing kidney and/or any other chronic health condition. A sum of the number chronic conditions was used for comparison between caregivers and non-caregivers.

Participants were asked questions about their satisfaction with care, ability to access care, importance of health care coordination and desire for access to additional health care services.

#### Satisfaction with care

Participants were asked “Overall, how satisfied are you with your personal health care experience?” and rated their responses on a 5-point scale ranging from ‘very satisfied’ to ‘very dissatisfied’.

#### Access to services

Difficulty in accessing health care and social services were measured on 6 domains. Participants were asked: “When it comes to your health care experience, how easy or difficult is it to: (1) see your primary health care provider when you need to, (2) Get mental health care or behavioral health services when you need them, (3) Get dental services when you need them, (4) Get emergency care when you need it, (5) Get medications if you need them and (6) Get health care and supportive services at home?” Participants rated their health care experience on a 5-point scale that ranged from ‘very easy’ to ‘very difficult’.

#### Coordination of services

Participants were asked to rate the importance of receiving home-based care and coordinating health care services when addressing their health care needs. The question asked: “When it comes to your personal health care experience, how important is (1) having a professional whose job it is to coordinate all aspects of your care, (2) having a way to receive health care services from your own home and (3) having a long-term relationship with your primary health care provider?” Responses were rated on a 5-point scale that ranged from ‘not at all important’ to ‘extremely important’.

#### Access to additional type of services

Participants were asked whether access to additional health care services would be important to their health care experience on 4 domains. Specifically, “In addition to receiving health care at doctor’s offices, clinics, and hospitals, how helpful would having access to (1) House calls, where your health care provider visits you in your home; (2) telemedicine, where you can receive care from your healthcare provider by phone or Skype or some other electronic device, (3) web portals on a computer or mobile device where you can log in online to communicate with your health care provider and view your personal health records in a secure environment and (4) services that deliver both prescriptions and over-the-counter medications to you in your own home.” Participants rated the helpfulness of these services on a 5-point scale ranging from ‘extremely helpful’ to ‘not helpful at all’.

#### Caregiver’s perceived support when caring for older adult

Finally, caregivers were asked about the level of support they receive in caring for their care recipient. Specifically, caregivers were asked: “Did you have the support that you need to provide ongoing living assistance to your older family member or friend? Would you say you had...” Caregivers chose between 5 response options when answering this question that ranged from ‘all the supports you needed’ to ‘none of the support you needed.’

### Data analysis and statistics

Survey weights were applied for all analyses. Descriptive statistics were used to calculate differences between caregivers and non-caregivers on demographics, satisfaction with care, access to services, coordination of services and access to additional type of services. A multivariate logistic regression model assessed correlates of caregivers not having the support they needed. Independent variables in the model included age, gender, education, income, ethnicity, region of country participant lives, number of chronic conditions, Medicaid status, physical disabilities, perceived quality of life, perceived finances and perceived health. All statistical analyses were conducted using GNU PSPP open source statistical analysis software release 0.10.4. A *p*-value of less than 0.05 was considered statistically significant.

## Results

A total of 3026 people completed the survey (76% via the web, 24% by telephone). The final stage completion rate was 45.4% (response rate of 21.3 and 94.4% weighted household panel retention).

A total of 1379 (45.8%) survey respondents reported a history of caregiving. Table 1 shows characteristics of caregivers and non-caregivers. Compared to non-caregivers, caregivers were more likely to be older (*p* < 0.001), female (*p* < 0.001), have lower educational attainment (*p* = 0.003), and live in a home owned by themselves or someone else in the household (*p* = 0.034). Caregivers, compared to non-caregivers were less likely to live in a metropolitan area (*p* = 0.002), married or living with a partner (*p* < 0.001), and have children living in the home (*p* < 0.001). Caregivers were more likely than non-caregivers to have multiple chronic health conditions (*p* < 0.001) and have a health condition or disability that impacts their daily life (*p* < 0.001).

**Table 1:** Characteristics of caregivers and non-caregivers

		Caregiver	Non-Caregiver	*p*-value
		(*n* = 1379)	(*n* = 1638)	
Age (mean, standard deviation)		57.0 (13.5)	50.3 (14.3)	<.001
% Female		59.5%	46.1%	<.001
Ethnicity				.030
Non-Hispanic White		68.8%	65.5%	
Black		11.6%	10.9%	
Hispanic		12.1%	15.8%	
Other		7.5%	7.8%	
Education				.003
No high school diploma		11.5%	10.3%	
High school graduate		31.0%	27.8%	
Some college		25.4%	23.3%	
Bachelor’s degree or above		32.1%	38.6%	
Employment status				<.001
Working		48.2%	61.4%	
Retired		29.9%	17.8%	
Not working		21.9%	20.8%	
Live in metropolitan area		85.0%	88.9%	.002
Home ownership		73.5%	69.4%	.034
Married or living with a partner		57.3%	64.1%	<.001
Children < 18 living in the household		23.6%	38.3%	<.001
Medicaid Insurance		17.8%	16.0%	.105
# Chronic Conditions				<.001
0		25.7%	42.3%	
1		27.9%	26.9%	
2		22.1&	16.7%	
3–4		20.6%	11.7%	
5 or more		3.9%	2.36%	
Have health conditions/physical disabilities that impact daily life/limit activities		34.9%	24.5%	<.001

Table 2 shows differences between caregivers and non-caregivers on their satisfaction with their care and perceived access to care for their personal health care experience. Caregivers (63.3%) were more satisfied with their personal health care experience compared to non-caregivers (59.4%; *p* = 0.03). Compared to non-caregivers, caregivers were more likely to report difficulty obtaining or getting mental health services (*p* = 0.006), dental services (*p* < 0.001), medications (*p* = 0.009), and supportive services as home (*p* < 0.001).

**Table 2:** Caregivers and non-caregiver’s perceptions and access to health care services

		Caregiver	Non-caregiver	*p*-value
Overall, how satisfied are you with your personal health care experience? (% very & somewhat satisfied)		63.3%	59.4%	.03
When it comes to your personal health care experience, how difficult is it to ….? (% somewhat to very difficult)				
• See your primary health care provider when you need to		18.7%	18.5%	.921
• Get mental health care or behavioral health services when you need them		29.4%	23.5%	.006
• Get dental services when you need them		23.8%	18.1%	<.001
• Get emergency care when you need it		9.6%	9.6%	.962
• Get medications if you need them		13.8%	10.6%	.009
• Get healthcare and supportive services at home		28.2%	18.7%	<.001
When it comes to your personal health care experience, how important is each of the following? (% very to extremely important)				
• Having a professional whose job it is to coordinate all aspects of your care		46.0%	36.7%	<.001
• Having a way to receive healthcare services from your own home		42.5%	32.8%	<.001
• Having a long-term relationship with your primary health care provider		68.1%	53.0%	<.001
In addition to receiving healthcare at doctor’s offices, clinics, and hospitals, how helpful would having access to each of the following services be to you? (% very to extremely helpful)				
• House calls		45.8%	35.6%	<.001
• Telemedicine		43.9%	39.2%	.009
• Web portals		49.6%	46.8%	.125
• Service that delivers medications to your home		56.5%	44.6%	<.001

For the question asking about coordination of services, caregivers were more likely than non-caregivers to say it was very important to have a care coordinator (*p* < 0.001), receive healthcare services in your home (*p* < 0.001), and have a long-term relationship with your primary care provide (*p* < 0.001). Finally, when asked about access to additional health care services, a greater proportion of caregivers, compared to non-caregivers, responded it would be very helpful to have access to house calls (*p* < 0.001), telemedicine (*p* < 0.001), and medications delivered to the home (*p* < 0.001).

When caregivers were asked if they had the support they needed to provide ongoing living assistance to their care recipient, 19% reported they had ‘hardly any’ or ‘none’ of the support they needed. In a multivariate logistic regression model (Table 3) correlates of not having support were younger age (OR = 0.67; *p* = 0.039), being Hispanic (OR = 1.58; *p* = 0.036), living in the western region of the US (OR = 1.64; *p* = 0.012), having more chronic conditions (OR = 1.25; *p* < .001), and more negative rating of the condition of personal household finances (OR = .1.37; *p* = 0.001).

**Table 3:** Correlates of caregivers not having support needed to provide ongoing care

	Adj OR	Lower CI	Upper CI	*p*-value
Age (30 to 64 years old ref)				
64 to 89 years old	.67	.46	.98	.039
Gender (Male ref)				
Female	.76	.56	1.05	.093
Ethnicity (white ref)				
Black	.53	.30	.96	.035
Hispanic	1.58	1.03	2.44	.036
Other	1.07	.62	1.86	.801
Income	1.03	.88	1.22	.707
Region (South ref)				
Northeast	.95	.59	1.51	.819
Midwest	1.16	.77	1.76	.471
West	1.64	1.12	2.41	.012
Medicaid	1.05	.70	1.56	.818
Perceived Health	.92	.75	1.13	.439
Health condition or physical disability	.97	.69	1.37	.860
# Chronic conditions	1.25	1.11	1.41	<.001
Rate household finances	1.37	1.14	1.64	.001

## Discussion

In this study, we aimed to identify how caregiving experience influences ability to reach health care services and whether having access to coordinated care or health services in the home would be beneficial. Additionally, we aimed to identify factors that contribute to caregiver having enough support when providing care. Nearly half of the national sample of US adults aged 30 to 89 years old were caregivers as defined with self-report of providing ongoing living assistance to an older family member or close friend. Family caregivers in our sample were more likely to be older, female, less educated, have more chronic conditions and physical disabilities than non-caregivers As hypothesized, family caregivers had more difficulty accessing and obtaining services, valued care coordination and wanted health care services in the home for their care compared to non-caregivers. Age, ethnicity, chronic conditions and confidence in household finances were factors that influenced whether the caregiver felt they had the support needed to assist their older care recipient.

Access to health care is complex and patient interactions with the health system are influenced by numerous factors. The conceptual framework of health care access [23] five defines dimensions of accessibility and our survey focused on the dimensions of Availability and accommodation and Appropriateness. Our results suggest health care access was influenced with the experience of caregiving with caregivers having more difficulty physically reaching timely access of health care services (Availability and accommodation) and engaging in services that fit their needs (Appropriateness). Although not directly measured in this survey, Approachability (knowledge health care service is available), Affordability (ability to pay for services) and Acceptability (cultural and social factors influencing access) could also be reasons for caregivers identifying barriers in the ability to seek, reach and engage in care. These barriers may be preventing caregivers from addressing their own health care needs.

Family caregivers had more difficulty accessing services and were more interested in health care services delivered in the home, such as telehealth and house calls, for their own personal health care than non-caregivers. These results are not surprising since the responsibilities and demands of caregiving may limit caregiver’s ability to attend appointments during business hours and obtain medications when needed suggesting barriers exist in Availability and accommodation domain. Home-based primary care or house calls provide high-quality care in the home for patients who have multiple comorbidities and functional limitations [24–27]. This medical model has been shown to increase satisfaction with care [25] and reduce burden or needs of caregivers [26, 27]. Similarly, the use of telehealth combined with coordinated health care services have been shown to decrease healthcare utilization and increase patient satisfaction in the Veteran’s Health Administration system [30, 31]. The use of telehealth for depression treatment has been shown to have similar outcomes to in-clinic treatment suggesting it may be a viable option for addressing behavioral health needs [32]. For caregivers juggling the responsibilities of caring for the care recipient and managing their own health needs, accessing medical care or behavioral health care in the home could be beneficial and potentially enable timely access to health care.

The fit of services to the patient need that allow for continuity and coordination of health care and allow patients to fully engage in their care describe the Appropriateness domain of conceptual mode of health care access. Our results suggest caregivers may have different needs for this domain than non-caregivers. Specifically, a long-term relationship with their primary care provider and a person to coordinate all aspects of their health care experience was important for caregivers. This view is consistent with previous research that found caregivers and patients with chronic disease have reported care coordination as a top priority when describing an ideal health care system [21]. The desire for effective communication and health care coordination may be influenced by the family caregiver’s experience of dealing with the health care activities of their care recipient with multiple chronic conditions [2] and the fragmentation of communication of care that often occurs across mu [33]. Family caregivers often must serve as the primary source of medical information for the care recipient [18, 33] within the health system and this role can inflict burden and stress on the caregiver. Family caregivers may benefit and engage in their health care when provided with patient-centered, coordinated care models that promote decision-making and reduce fragmented medical care [26] particularly the caregivers in our sample who experience multiple chronic conditions and functional limitations.

Caregivers in our study were more satisfied with their health care experience than non-caregivers but had more difficulty accessing services such as dental, behavioral health and medications. No significant differences were found between caregivers and non-caregivers when asked about access to their primary care provider or emergency care. We speculate that caregivers may be considering health care experience as their experience with their primary care provider when rating satisfaction with care. It is also possible that caregiver’s difficulty in accessing medications, dental services and behavioral health may be due to financial constraints associated with these services such as dental insurance, co-pays or medication costs. As described in the conceptual model, Affordability is related to direct costs and these barriers can limit ability to access health care services. The result of high satisfaction with health care experience but greater difficulty accessing services suggests that these survey questions are addressing different determinants of access to health care services. However, further in-depth questions would be necessary to extrapolate these findings.

Caregivers who reported little to no support in providing ongoing living assistance for their older family member or friend were more likely to be younger, have chronic conditions, have negative view of finances, live in Western U.S. and Hispanic ethnicity. Lack of informal support or support from family or friends is a critical component to consider for health care providers since it has been associated with worse physical health in the caregiver [7]. In our sample, it’s possible that lack of support in younger caregivers (aged 30–65 years old) may be due to the conflicting demands on their time and resources than older caregivers such as employment and parenting of children [34]. The added responsibilities of caring for older care recipient could contribute to financial stress which has been associated with increased caregiver burden [19].

Caregiving can be physically and emotionally demanding [1, 5, 35] and the presence of chronic conditions could limit the ability of the caregiver to meet the needs of their care recipient resulting in lack of feeling the caregiver has necessary support. Community programs have been shown to offer support and reduce caregiver burden [14, 36] but it’s possible that younger caregivers and caregivers with chronic conditions are unaware of these programs or do not have the ability or time to participate in these programs. Similarly, if community programs do not address the differences in culture of individuals providing care, it may not be effective in reducing burden or providing support. Cultural differences in approaches to caregiving and the disproportionate impact of caregiving, especially for Hispanic caregivers, have been described in the literature [9, 37–39]. Within Hispanic caregivers, a recent study found Hispanic caregivers provided more caregiver hours per week, used respite care the least and provided longer duration of caregiving when compared to previous generations of Hispanic caregivers [40]. Similarly, Hispanic caregivers of individuals with dementia who reported high burden were found to have depressive symptoms, low satisfaction with their social networks and greater co-morbidities [41].

The limitations with our research study are similar to other survey research designs. The cross-sectional design of the study precludes understanding the causality or direction of the associations found in the data. While the survey sample was weighted to be representative of US adults aged 30 to 89 years old, it may underrepresent individuals who are uninsured or unhealthy. We did not have a way to validate the self-reported measure that classified respondents into caregivers or non-caregivers. Caregivers in our study were identified as ever offering assistance to an older adult. This broad definition may have introduced bias and is different from other studies that typically ask if individual has provided assistance in the past month [3]. The intensity of caregiving and the duration of caregiving was not assessed in this survey which may have added bias to the sample. Strengths of the study include a large representative sample of US adults and multiple measures of perceptions of access to health and community services.

Though the results of this study are promising, they could be strengthened by collecting qualitative data to better understand the caregivers’ perspective about challenges tending to their own personal health while simultaneously being a caregiver. Specifically, using qualitative data to complement the survey findings could help clarify barriers to accessing behavioral health, medications, dental services and supportive services in the home. Future research should focus on the identifying access to care barriers, as well as the health and well-being impacts of alleviating these barriers with innovative care models, such as coordinated care, home-based care or medication home delivery—for both the care recipient and the caregiver.

## Conclusions

In this cross-sectional study design, we have identified how caregiver experience influences access to health care and identified how access to additional health care services would be valuable to caregivers in addressing their health care needs. Caregivers were in poorer health than non-caregivers in our sample and had trouble accessing medical, dental and behavioral health care as well as supportive services. The difficulty in accessing care suggests caregivers value services delivered in the home such as telehealth and home-based primary care. Caregivers also valued long-term relationship with health care provider and a person to coordinate all aspects of their care than non-caregivers. These results reinforce research on caregiver stress and burden but at a national level. One of the more interesting findings of this work is that caregivers were more satisfied with their own care than non-caregivers but had more trouble accessing services. These results provide evidence on access to care differences for caregivers and identify health care models that may help to broaden access to care.

## Data Availability

The datasets generated and/or analyzed during the current study are not publicly available due participant confidentiality but are available from the corresponding author on reasonable request.

## Abbreviation

NORC: National Opinion Research Center
